# Chemokine/ITGA4 Interaction Directs iPSC-Derived Myogenic Progenitor Migration to Injury Sites in Aging Muscle for Regeneration

**DOI:** 10.3390/cells12141837

**Published:** 2023-07-12

**Authors:** Muhammad Ashraf, Srinivas M. Tipparaju, Joung Woul Kim, Wanling Xuan

**Affiliations:** Department of Pharmaceutical Sciences, USF Health Taneja College of Pharmacy, University of South Florida, Tampa, FL 33612, USA; ashrafm206@usf.edu (M.A.); stippara@usf.edu (S.M.T.); joungwoulkim@usf.edu (J.W.K.)

**Keywords:** ITGA4, chemokines, migration, muscle progenitor cell, muscle injury, sarcopenia, aging

## Abstract

The failure of muscle to repair after injury during aging may be a major contributor to muscle mass loss. We recently generated muscle progenitor cells (MPCs) from human-induced pluripotent stem-cell (iPSC) cell lines using small molecules, CHIR99021 and Givinostat (Givi-MPCs) sequentially. Here, we test whether the chemokines overexpressed in injured endothelial cells direct MPC migration to the site by binding to their receptor, ITGA4. ITGA4 was heavily expressed in Givi-MPCs. To study the effects on the mobilization of Givi-MPCs, ITGA4 was knocked down by an ITGA4 shRNA lentiviral vector. With and without ITGA4 knocked down, cell migration in vitro and cell mobilization in vivo using aged NOD scid gamma (NSG) mice and mdx/scid mice were analyzed. The migration of shITGA4-Givi-MPCs was significantly impaired, as shown in a wound-healing assay. The knockdown of ITGA4 impaired the migration of Givi-MPCs towards human aortic endothelial cells (HAECs), in which CX3CL1 and VCAM-1 were up-regulated by the treatment of TNF-α compared with scramble ones using a transwell system. MPCs expressing ITGA4 sensed chemokines secreted by endothelial cells at the injury site as a chemoattracting signal to migrate to the injured muscle. The mobilization of Givi-MPCs was mediated by the ligand–receptor interaction, which facilitated their engraftment for repairing the sarcopenic muscle with injury.

## 1. Introduction

Sarcopenia is characterized by muscle mass loss and function decline with age, and is a major concern for lifelong health and well-being in the elderly population, regardless of injury. Weaker muscles with lower mass and function are likely more susceptible to muscle damage and injury [1]. No effective cure is yet available for muscle loss during aging [2]. Although pharmacologic approaches have been proposed to combat sarcopenia, these are not without potential risks and secondary effects [3]. Exercise has been considered an appropriate approach to combat sarcopenia; however, patients with underlying health conditions or patients with muscle injury may not adhere to exercise or physical therapy, rendering the need for alternate therapy [4]. After severe damage, muscles in aging rodents did not regenerate [5,6,7,8]. At the same time, of paramount importance and necessity is the search for effective cell types that favor skeletal muscle regeneration. Muscle stem cells (MuSCs) are major myogenic progenitors for adult muscle homeostasis and repair [9]. Dysregulation of the MuSC pool may contribute to the accelerated loss of skeletal muscle mass that is observed with advancing age [10,11].

Stem-cell-based therapy holds great promise to restore adequate skeletal muscle structure and function in the elderly with sarcopenia, especially under injury conditions [2]. However, it is plagued with issues of deliverability and in vitro expansion [12,13]. Meanwhile, there have been few studies involving human stem-cell transplantation in aging preclinical animal models. Technological advances in reprogramming individuals’ somatic cells into iPSCs have provided a potential alternative for replacing dysfunctional muscle tissue [14,15]. Senescent and centenarian iPSCs can be re-differentiated into fully rejuvenated cells [16]. Importantly, iPSCs are also easily converted into muscle progenitor cells (MPCs) with the overexpression of myogenic transcription factors using virus vectors or small molecules [15,17] and these are easily expandable and do not induce an immune response or tumor formation upon transplantation. However, stem-cell-based approaches for skeletal muscle regeneration are plagued by the poor engraftment of progenitors in harsh aging microenvironments. In our previous study [14], we successfully generated muscle progenitor cells (MPCs) from multiple human iPSC cell lines using small molecules CHIR99021 and Givinostat. The Givinostat-induced MPCs (Givi-MPCs) develop into new muscle fibers upon transplantation into the injured muscles of dystrophy mice. During aging, increased inflammation and oxidative damage in skeletal muscle tissues occur. Thus, cell-based therapy in aging muscle, especially in injured aging muscle, remains a challenge. Furthermore, it is known that myogenic progenitor cell migration is crucial for promoting rapid tissue regeneration [18,19]. We have demonstrated that Givi-MPCs possess anti-oxidative and anti-inflammatory properties and exhibit superior migration characteristics and a high expression of the chemokine receptor ITGA4 in these cells [14]. Here, we demonstrate that chemokine/the ITGA4 chemotactic pathway directs iPSC-derived myogenic progenitor cell migration to injury sites in dystrophic muscle. We found that Givi-MPC migration to injured muscle sites is responsible for improved muscle regeneration, both in aged NOD scid gamma (NSG) mice and mdx/scid mice (a mouse model for Duchenne muscular dystrophy).

## 2. Materials and Methods

### 2.1. Human iPSC Culture

The human iPSC cell line DYS0100 from the ATCC Company was used. For in vivo cell-tracking, the H2B-EGFP reporter iPSCs cell line (AICS-0061) from the Allen Institute, Seattle, WA, USA was also used for differentiation. iPSCs were cultured on a vitronectin-coated six-well plate in a mTeSR1 medium (STEMCELL Technologies, Vancouver, BC, Canada) with daily medium change. iPSCs were passaged using ReLeSR™ passaging reagent (STEMCELL Technologies, Vancouver, BC, Canada).

### 2.2. Generation of iPSC-Derived Muscle Progenitor Cells

Muscle progenitor cells (MPCs) were generated using Givinostat, as described in our previous study [14]. Briefly, iPSCs at 3 × 10^5^ cell/well density in a six-well plate were cultured in mTeSR1 medium with 5 μM ROCK inhibitor (Y-27632, STEMCELL Technologies, Vancouver, BC, Canada) for 24 h followed by treatment with CHIR99021 (10 μM) in the E6 medium (Thermo Fisher Scientific, Waltham, MA, USA) for 2 days and then with Givinostat (100 nM) for 5 days. Fourteen days after differentiation, Givinostat-induced MPCs (Givi-MPCs) were expanded in an SKGM-2 medium (Lonza, Morristown, NJ, USA) plus FGF-2 (2.5 ng/mL), and cells at passages 2–4 were used for experiments.

### 2.3. Generation of ITGA4 Knockdown Muscle Progenitor Cells

To study the effects of ITGA4 on mobilization and engraftment, stable ITGA4 knockdown Givi-MPCs were generated by the transduction of human ITGA4 shRNA lentivirus vector and puromycin selection. The control Givi-MPCs were transduced with a scrambled shRNA lentivirus vector. Lenti-H1-human ITGA4 shRNA-CMV-GFP-2A-Puro virus and Lenti-H1-scrambled shRNA-CMV-GFP-2A-Puro virus were obtained from Applied Biological Materials Inc., Richmond, BC, Canada.

shRNA sequences are listed below:

Human ITGA4 shRNA: CCGGGCTCCGTGTTATCAAGATTATCTCGAGATAATCTTGATAACACGGAGCTTTTT;

Scrambled shRNA: CCGGTAGCGACTAAACACATCAATCCTCGAGGATTGATGTGTTTAGTCGCTATTTTTG.

Givi-MPCs (50–60% confluence) were transduced with ITGA4 shRNA lentivirus vector or scramble vector at an MOI of 2. After 48 h of transduction, the selection of transduced cells was made by the addition of 1 μg/mL puromycin, which was sufficient for the efficient killing of non-transduced cells, resulting in almost alive GFP-positive Givi-MPCs (Supplementary Figure S1).

### 2.4. Cell Migration

With a wound-healing assay, Givi-MPCs with or without ITGA4 silencing were seeded into a 35 mm dish with culture-insert 2-well (ibidi, Fitchburg, MA, USA) at 1 × 10^5^/mL concentration in an SKGM-2 medium with 2% fetal bovine serum (FBS). The next day, a confluent layer was observed, culture inserts were removed, and after 24 h, the number of migrated cells was analyzed. A transwell system was used to test the role of ITGA4 in Givi-MPC chemotaxis towards endothelial cells with inflammatory stimulation. The HAECs were cultured in the lower chamber with tumor necrosis factor-α (TNF-α) pretreatment. Givi-MPCs with or without ITGA4 silencing were seeded in the upper chamber. The number of migrated cells was analyzed by the number of GFP-positive cells in the lower chamber. In addition, a monolayer of HAECs with TNF-α exposure using a Boyden chamber was used to mimic the in vivo inflammatory environment, and the number of Givi-MPCs across the monolayer HAECs was counted.

### 2.5. Immunofluorescence Staining for Cells

Cells were fixed with 4% PFA for 10 min and blocked with 10% FBS for 1 h at room temperature. Cells were incubated with primary antibodies including anti-CD49/ITGA4 (1:100, Novus Biologicals, Centennial, CO, USA) and Ki67 (1:300, Abcam, Cambridge, UK), respectively, at 4 °C overnight, and a secondary antibody was conjugated to Alexa Fluor 594 (Life Technologies, Carlsbad, CA, USA) at room temperature for 1 h. Images were taken using a fluorescent microscope (Olympus, Tokyo, Japan).

### 2.6. RNA Extraction and Realtime-PCR

Total RNA was isolated using the RNeasy Mini Kit (Qiagen, Hilden, Germany). Reverse transcription was performed using the QuantiTect Reverse Transcription kit (Qiagen, Hilden, Germany). qRT-PCR was performed on the Q3 real-time PCR machine (ABI) using the Quantitate SYBR Green real-time PCR method as described elsewhere. Primer sequences are as follows: CX3CL1: forward 5′-TCCACTATCAACAGAACCAGGCATC-3′; reverse 5′-TCGGGTCGGCACAGAACAGC-3′; vascular cell adhesion molecule 1 (VCAM-1): forward 5′-GCAAGGTTCCTAGCGTGTAC-3′, reverse 5′-CAATGGTAGGGATGAAGGTC-3′; GADPH: Forward 5′-TGCACCACCAACTGCTTAGC-3′, reverse 5′-GGCATGGACTGTGGTCATGAG-3′ act as the loading control. The fold change of expression level for each gene was determined by the expression 2^−∆∆CT^. The final values were averaged, and results were represented as fold expressions with the standard deviation (SD).

### 2.7. Chromatin Immunoprecipitation (ChIP)

Chromatin immunoprecipitation assay was performed using the Chromatin Extraction Kit (Abcam) and Pierce Magnetic ChIP Kit (Thermo Fisher Scientific, Waltham, MA, USA) according to the manufacturer’s instructions. Briefly, cells were incubated with 37% formaldehyde to cross-link proteins to DNA. Then, glycine was used to stop the formaldehyde reaction. Cells were lysed and sonicated on ice for 30 s to obtain soluble sheared chromatin (average DNA length of 200–500 bp). A total of 1% of chromatin was stored at −20 °C as input DNA, and the rest was utilized for immunoprecipitation. Immunoprecipitation was achieved using the following antibody against anti-H3K27ac (Cell Signaling Technology, Danvers, MA, USA). The beads were mixed with chromatin extracts of cells and rotated overnight. The immunoprecipitated DNA was purified using a DNA isolation buffer. Quantification of ChIP-enriched DNA and the relative levels of DNA of ITGA4 promoters were analyzed by real-time PCR. The detailed information for the ITGA4 ChIP primers is as follows: ITGA4-1 ChIP-F1 5′-TGCCTACACCTGAAAAACAAG-3′; ChIP-R1 5′-GGCTCCGTCTCTGCCTAC-3′. ITGA4–2: ChIP-F1 5′-GCACATTTCAGAGGCTCATTA-3′; ChIP-R1 5′-TGGGGAACATTTTAGTGACAA-3′. The IP/INPUT ratio of the target sequence was measured using the following formula: (% IP/INPUT = 2[(Ct (x % input) − log (x %)/log2) − Ct (IP)] × 100). The fold enrichment was normalized to non-immune IgG signals.

### 2.8. Muscle Injury Model and Cell Transplantation

Animal experiments were carried out according to the experimental protocol approved by the Augusta University Animal Care and Use Committee and the University of South Florida Animal Care and Use Committee. Three-month-old and eighteen-month-old NSG mice (Stock No: 005557, The Jackson Laboratory, Bar Harbor, ME, USA), and sixteen-month-old mdx/scid mice (Stock No: 018018, The Jackson Laboratory, Bar Harbor, ME, USA) were used in the present study. Mice were anesthetized using 2% isoflurane, and the right tibialis anterior (TA) muscle was injured with 50 μL of 10 μM cardiotoxin (Naja mossambica-mossambica, Sigma-Aldrich, Inc., St., Louis, MO, USA). After 24 h, shITGA4-Givi-MPCs were dissociated using Accutase (STEMCELL Technologies, Vancouver, BC, Canada) and resuspended in Dulbecco’s phosphate-buffered saline (DPBS) at 5 × 10^5^ per 200 μL. Cells were injected through the tail vein. For local cell transplantation, Givi-MPCs differentiated from H2B-EGFP-iPSC at 1 × 10^5^ per 20 μL were transplanted into the TA muscle by intramuscular injection. For each experimental model and time point, 10–12 mice were analyzed, and both male and female mice were used in separate groups. No mice were excluded during the analysis. At the endpoint, mice were euthanized with inhalation overdose of isoflurane (5%), or CO_2_ followed by bilateral thoracotomy instead of pneumothorax before subjecting them to tissue removal. The reporting of animal experiments in this study adheres to the ARRIVE guidelines.

### 2.9. Histology

TA muscle tissue was harvested and processed for hematoxylin and eosin (HE), Sirius red, and Masson’s trichrome staining, as reported previously [14]. Fibrosis was determined using the ImageJ software (NIH), as previously described [14].

### 2.10. Immunofluorescence Staining for Muscle Tissue

TA muscles were harvested and fixed with 4% paraformaldehyde (PFA) for 1 h at room temperature and then immersed in 30% sucrose overnight at 4 °C, as described in our previous study [14]. On day 2, the TA muscles were cryopreserved in an optical cutting temperature (OCT) compound (Tissue Tek) at −80 °C. TA muscle samples were sliced into 5-μm-thick frozen cross-sections using a Leica CM3050 cryostat. The muscle sections were incubated with primary antibodies including human-specific dystrophin (NBP2-79783, 1:200, Novus Biologicals, Centennial, CO, USA), human nuclear antigen (NBP2–34342, 1:100, Novus Biologicals, Centennial, CO, USA), CX3CL1(1:100, ABclonal, Woburn, MA, USA), VCAM-1(1:100, ABclonal, Woburn, MA, USA) and Myh3 (DSHP, 1:100) at 4 °C overnight, respectively, and anti-rabbit/mouse/rat secondary antibodies conjugated to Alexa Fluor 594 (Life Technologies) at room temperature for 1 h. Images were taken using a confocal microscope (FV1000, Olympus, Tokyo, Japan). For cell-engraftment quantification, four sections at 150 μm intervals in each TA muscle were analyzed. The number of muscle fibers in a cross-section area was measured using ImageJ with the colocalization plugin (NIH).

### 2.11. Grip Strength Test

The hindlimb grip strengths were measured with the use of a Chatillon force measurement DFE II grip strength meter (Ametek, Columbus Instruments, Columbus, OH, USA) [20]. The force measurement was recorded until the mice were released from the T-bar. Five repetitions for each mouse were recorded. The normalized grip strength was calculated by dividing the mean grip strength by each mouse’s body weight (n = 5).

### 2.12. Statistical Analysis

Data were expressed as mean ± SD. Normality was tested, and the statistical analysis of differences among the different groups was compared by unpaired two-tailed Student’s *t*-tests. The differences were considered statistically significant at *p* < 0.05. Statistical analyses were performed by GraphPad Prism 6.0 (Chicago, IL, USA).

## 3. Results

### 3.1. In Vitro and In Vivo Migration Characteristics of Givi-MPCs

**Figure 1:**
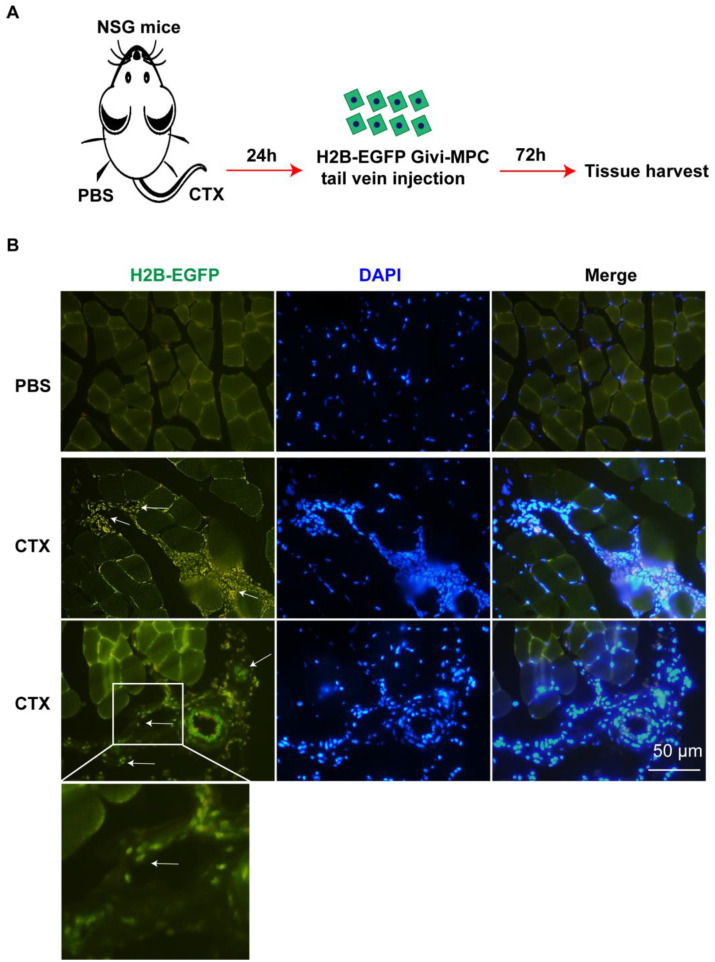
Tracking of Givi-MPCs by systemic delivery. (**A**) Schematic protocol for Givi-MPCs delivery by intravenous injection. (**B**) Representative images of tracked cells with H2B-EGFP fluorescence in CTX-injured muscle or PBS-treated control muscle tissue sections. White arrows point towards transplanted cells. Bar = 50 µm. n = 6.

**Figure 2:**
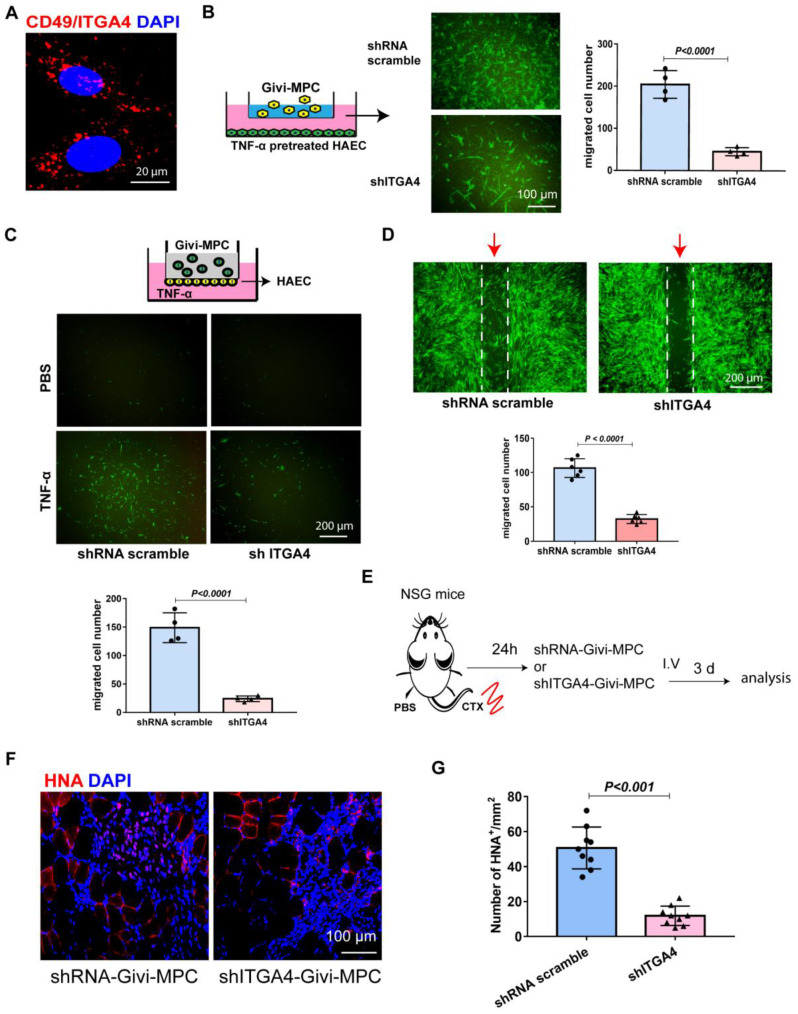
ITGA4 (CD49) directs migration and mobilization of Givi-MPCs. (**A**) Representative immunostained image of ITGA4 (CD49) expression in Givi-MPCs. (**B**) Schematic diagram of transwell system for determining Givi-MPCs migration towards inflammatory human aortic endothelial cells (HAECs). Representative GFP fluorescence image of migrated Givi-MPC transfected with either shRNA scramble or shITGA4 in the bottom chamber. Semi-quantitative estimate of Givi-MPCs with or without ITGA4 silencing. Bar = 100 µm. (**C**) Schematic diagram of a Boyden chamber for determining the Givi-MPC migration through a monolayer of HAEC under the inflammatory environment. Representative GFP fluorescence image of migrated Givi-MPCs with either shRNA scramble or shITGA4 transfection in the bottom chamber and quantitative estimate of migrated cells. Bar = 200 µm. (**D**) Representative images and quantitative estimate of cell migration by shRNA-Givi-MPC and shITGA4-Givi-MPCs (arrows). Bar = 200 µm. (**E**) Schematic protocol outline for shRNA-Givi-MPCs or shITGA4-Givi-MPCs delivery by intravenous injection. (**F**) Representative images of human nuclear antigen (HNA) expression in CTX-injured muscle tissue after 3 days of transplantation in old NSG mice with either shRNA-Givi-MPCs or shITGA4-Givi-MPCs by IV injection. Bar = 100 µm. (**G**) Semi-quantitative estimate of HNA-positive cells in CTX-injured muscle tissue from mice transplanted with Givi-MPCs with or without ITGA4 silencing. Quantitation data were derived from nine muscle sections of three NSG mice in each group.

**Figure 3:**
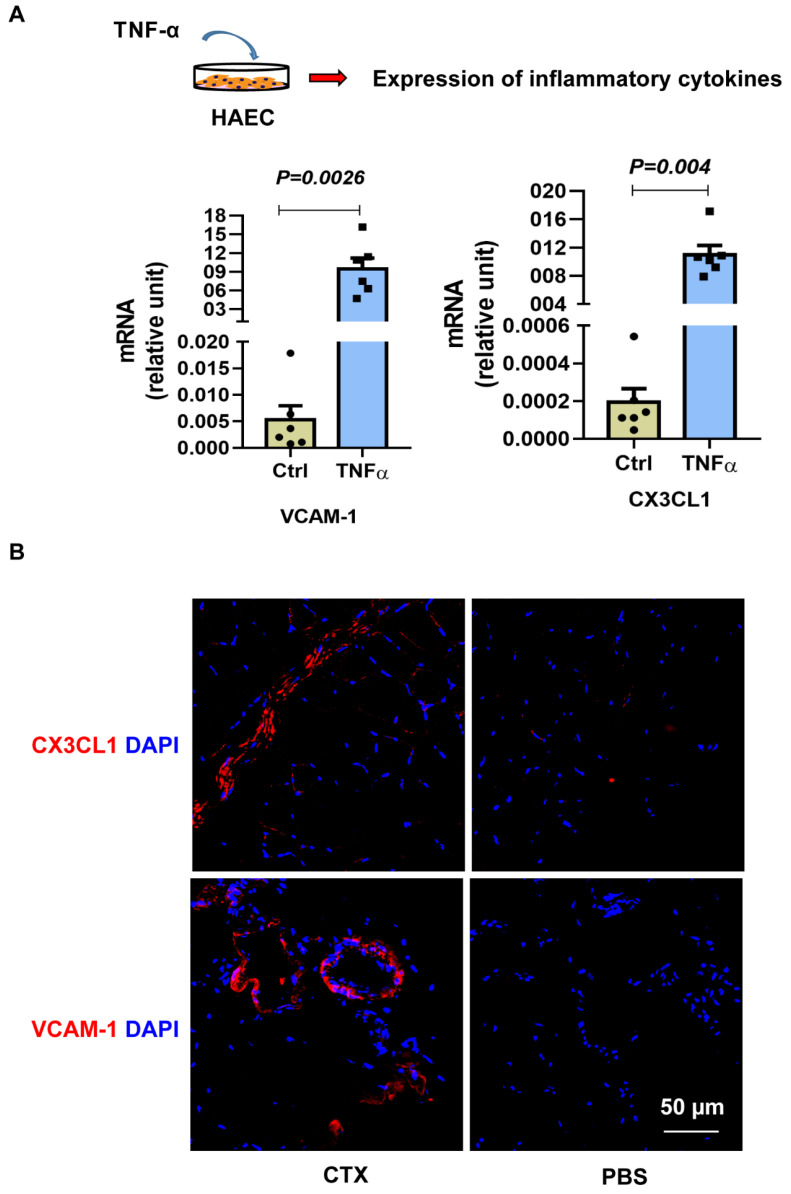
Increased ITGA4 ligand expression in endothelial cells. (**A**) Expression of ITGA4 ligands: VCAM-1 and CX3CL1 in HAECs with or without TNF-α treatment by real-time PCR. (**B**) Representative fluorescent image of CX3CL1 and VCAM-1 in CTX-injured muscle tissue. Bar = 50 µm. n = 6.

Muscle atrophy is a common problem in aging. The muscle injury is not localized, but randomly spread out within the tissue. For stem-cell-based therapy, the mode of delivery is very important, particularly in muscular dystrophy disease. Accordingly, we explored whether Givi-MPCs could target injured muscle tissue upon systemic administration in aging mice. To track the Givi-MPCs after systemic delivery, we differentiated Givi-MPCs from the H2B-EGFP reporter iPS cell line. These Givi-MPCs were confirmed with the expression of myogenic transcription factor Pax7 (Supplementary Figure S2). Next, we induced muscle injury using CTX in the right TA muscle of 3-month-old NSG mice, and the left TA muscle was injected with PBS as a control. The Givi-MPCs derived from the H2B-EGFP reporter iPS cell line were injected via the tail vein. A total of 72 h after injection, TA muscle tissues were harvested for analysis, as outlined in Figure 1A. Interestingly, the transplanted Givi-MPCs (H2B-EGFP-positive cells with colocalization of the DAPI signal) were observed in the injured TA muscle tissue and not in PBS-treated TA muscle tissue (Figure 1B). Human nuclear antigen (HNA) staining also confirmed the identity of engrafted EGFP-positive Givi-MPCs in injured TA muscle tissue (Supplementary Figure S3). Moreover, these MPCs were not observed in other organs such as the heart, lung, liver, or kidney (Supplementary Figure S4). This observation supported our rationale that the systemically delivered Givi-MPCs were mobilized toward injured muscle tissue. Next, we asked the question of what drove the transplanted Givi-MPCs toward injured sites. We demonstrated a high expression of ITGA4 in Givi-MPCs both by PCR-array assay [14] and immunostaining (Figure 2A). At the same time, the chemokines, VCAM-1, and CX3CL1 were strongly up-regulated in endothelial cells during inflammation, and their receptor ITGA4 was expressed in Givi-MPCs. To support our rationale, we determined in vitro whether Givi-MPCs can migrate towards human aortic endothelial cells (HAECs), in which CX3CL1 was up-regulated by the treatment of an inflammatory cytokine TNF-α using a transwell system. To explore the role of ITGA4 in the mobilization/migration of Givi-MPCs, ITGA4 was silenced by the shRNA lentivirus vector. ITGA4 silencing impaired the migration of Givi-MPCs towards inflammatory cytokine TNF-α pretreated HAECs in the bottom chamber compared with scramble ones (Figure 2B). Furthermore, we exposed the monolayer of HAEC to the TNF-α using a Boyden chamber to mimic the in vivo inflammatory environment, and then tested whether the Givi-MPCs could cross the EC barrier. The setting is displayed in Figure 2C. Givi-MPCs with scramble vector transfection crossed the EC barrier under the inflammatory environment, as demonstrated by the GFP signal in the bottom chamber. However, a limited number of GFP-positive cells in the bottom chamber was observed from ITGA4-silenced Givi-MPCs (Figure 2C). Migration of shITGA4-Givi-MPCs was significantly impaired, as demonstrated by the migration of fewer cells in a wound-healing assay compared with Givi-MPCs transfected with scramble vector (Figure 2D). These results were consistent with the transplantation study, where fewer HNA-positive cells were seen in CTX-injured TA muscle after transplantation of ITGA4-silenced Givi-MPCs (tail-vein injection) (Figure 2E,F). We further confirmed an increased expression of VCAM-1 and CX3CL1 in HAECs after TNF-α stimulation by real-time PCR (Figure 3A). Enhanced expression of VCAM-1 and CX3CL1 was also observed in CTX-injured TA muscle tissue (Figure 3B). Taken together, these data demonstrated that inflammation enhanced the expression of chemokine ligands, which favored MPCs homing to chemokine-rich cellular sites towards the injured sites.

### 3.2. Knockdown of ITGA4 Impaired Proliferation and Differentiation of Givi-MPCs

**Figure 4:**
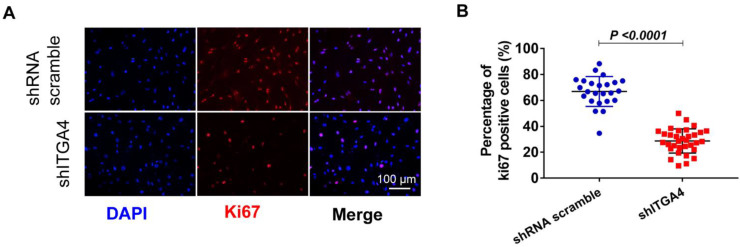
Knockdown of ITGA4 impaired proliferation of Givi-MPCs. (**A**) Representative fluorescent images of Ki67 expression in Givi-MPCs with or without ITGA4 silencing. Bar = 100 µm. (**B**) Percentages of Ki67 positive cells in Givi-MPCs with or without ITGA4 silencing.

Besides impaired mobilization of ITGA4 silenced Givi-MPCs, the proliferation of Givi-MPCs was also compromised, as demonstrated by a low percentage of Ki67 Givi-MPCs (Figure 4A,B), compared to the scramble ones.

### 3.3. Both Local and Systemic Delivery of Givi-MPCs Improved Muscle Regeneration in Aging Muscle

**Figure 5:**
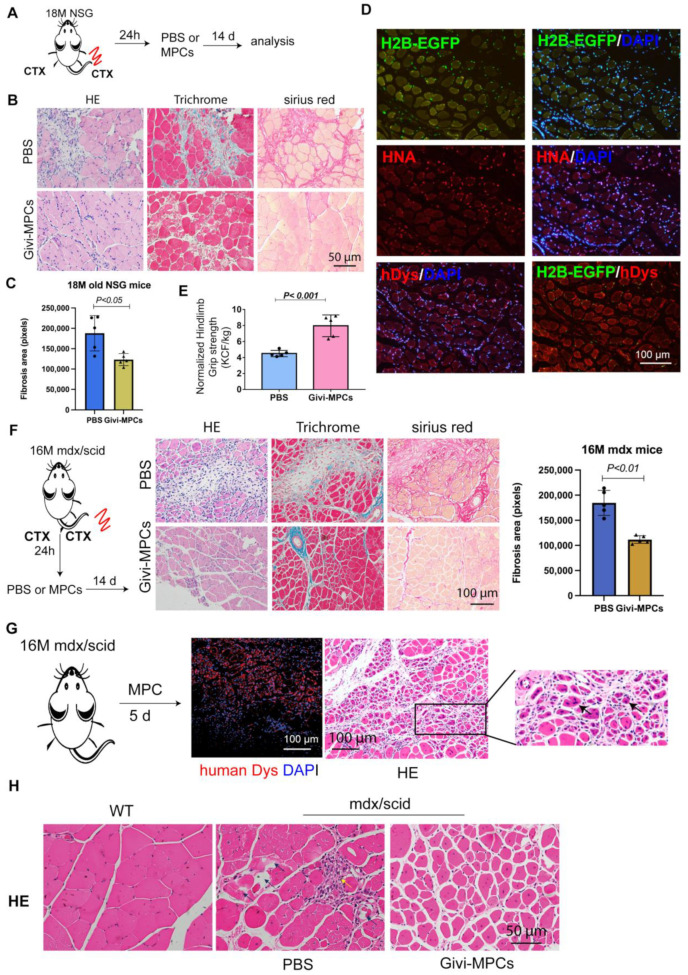
Improvement of muscle regeneration in aging muscle with Givi-MPCs transplantation. (**A**) Experimental outline of muscle injury and cell transplantation in 18 M old NSG mice. (**B**) Representative images of HE staining, Trichrome Masson staining, and Sirius red staining for TA muscle tissue from 18 M old NSG mice. Bar = 50 µm. (**C**) Quantification of fibrosis area in 18-month-old NSG mice with different treatments. (**D**) Representative fluorescence images for tracking EGFP-H2B cells, HNA, and human-specific dystrophin (hDys) expressing cells from tibialis anterior (TA) muscle tissue sections with Givi-MPCs transplantation. Bar = 100 µm. (**E**) Hindlimb grip strength improvement in 18-month-old CTX-injured NSG mice with Givi-MPC transplantation. *P* < 0.001. n = 5. (**F**) Experimental scheme for muscle injury and cell transplantation in 16-month-old mdx/scid mice. Representative images of HE staining, Trichrome Masson staining, and Sirius red staining for TA muscle tissue from 16-month-old mdx/scid mice. Bar = 100 µm. Quantification of fibrosis area in 16-month-old mdx/scid mice with different treatments. n = 5. (**G**) Representative images of HE staining and human dystrophin expression from non-injured 16-month-old mdx/scid mice 5 days after Givi-MPCs transplantation. Bar = 100 µm. Arrows point out the regenerating muscle cells with central nuclei. n = 5. (**H**) HE staining images of TA muscle tissue from 16-month-old mdx/scid mice either with PBS treatment or Givi-MPCs treatment for one month and age-match wild-type mice. Bar = 50 µm. The black arrows indicate muscle necrosis and the yellow arrows indicate infiltrated mononuclear cells. n = 6.

We tested whether superior migratory characteristics of Givi-MPCs resulted in improved muscle regeneration in aging mice upon injury. Givi-MPC_S_ were transplanted into TA muscle after CTX injury in 18-month-old NSG mice and 16-month-old mdx/scid mice. The contralateral TA muscle was injected with PBS as a control. For the grip strength study, mice were either injected with PBS or Givi-MPCs after CTX injury. Compared with PBS-treated TA muscle, limited infiltration of mononuclear cells, reduced muscle necrosis, and fibrosis were observed in Givi-MPCs transplanted TA muscle in 18-month-old NSG mice (Figure 5A–C). To determine the MPC identity after transplantation, we also directly injected Givi-MPCs induced from the H2B-EGFP iPS cell line into the TA muscle of old NSG mice. A total of 14 days after transplantation, MPCs detectable by H2B-EGFP signal, HNA-positive cells, and human dystrophin-positive cells were observed in the muscle tissues (Figure 5D), suggesting the engraftment of Givi-MPCs in injured aging muscle tissue. Importantly, the grip strength was significantly improved in mice with cell transplantation (Figure 5E). Similar findings were observed in 16-month-old mdx/scid mice with CTX injury, where Givi-MPCs transplantation reduced the inflammation and decreased muscle fibrosis (Figure 5F). We also tested the effect of Givi-MPCs in these mice without CTX injury (Figure 5G). Five days after transplantation, numerous small-size muscle cells with central nuclei were verified by HE staining (Figure 5G). Importantly, human-specific dystrophin-positive cells were prominently observed, suggesting a strong muscle regeneration in aging mdx/scid mice transplanted with Givi-MPCs (Figure 5G). One month after transplantation, muscle necrosis, and inflammation were also significantly reduced compared with PBS-treated TA muscle (Figure 5H).

**Figure 6:**
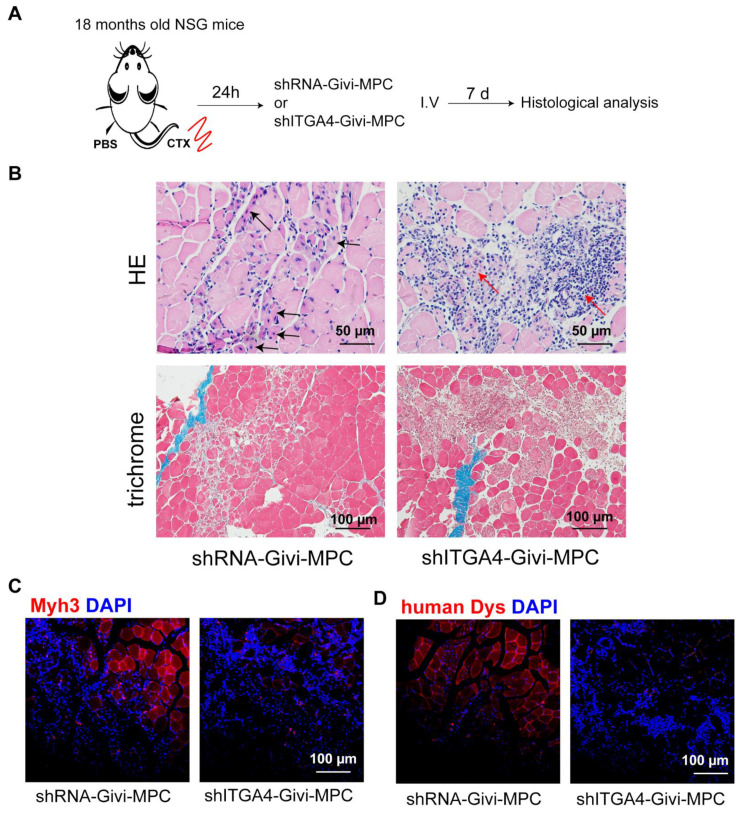
ITGA4 silencing impaired muscle regeneration in aging muscle with systemic delivery of Givi-MPCs. (**A**) Schematic protocol for Givi-MPC delivery by intravenous injection in aging mice. (**B**) Representative images of HE staining (Bar = 50 µm) and Trichrome Masson staining (Bar = 100 µm) for TA muscle tissue from 18-month-old aging NSG mice with shRNA-Givi-MPCs or shITGA4-Givi-MPCs transplantation by IV injection. Black arrows indicate regenerating muscle cells with central nuclei; red arrows indicate infiltrating mononuclear cells. Representative fluorescent images of Myh3 (**C**) and human dystrophin (**D**) expression in the muscle section. n = 6. Bar = 100 µm.

Finally, we investigated whether the systemic injection of Givi-MPCs led to their homing to the site of injury for engraftment and muscle regeneration in aging mice (Figure 6A). We carried out in vivo experiments to explore the behavior of Givi-MPCs by systemic delivery. The occurrence of embolism is one of the safety issues in the systemic delivery of cells [21]. In our study, we used a dosage of 5 × 10^5^ cells for tail-vein injection, and no embolism was observed with this dosage. However, with an increase in dosage to 1 × 10^6^ cells per injection, some mice (2/10) died of pulmonary embolisms, as demonstrated by the presence of EGFP-positive cells in the lumen of the pulmonary vessels (Supplementary Figure S5). Therefore, dosage optimization should be considered for systemic application. A small number of muscle cells with central nuclei were observed in injured TA muscle tissue of 18-month-old NSG mice treated with Givi-MPCs as compared to scramble-treated mice at 7 days post-injury. In addition, many mononuclear cells accumulated in the necrotic area of the mice treated with ITGA4-silenced Givi-MPC (Figure 6B). Immunofluorescence staining also revealed a higher expression of Myh3 (embryo myosin) and human dystrophin in aging NSG mice treated with Givi-MPCs compared to mice treated with ITGA4 silenced Givi-MPCs (Figure 6C,D). These data supported the view that Givi-MPCs delivered locally or systemically mobilized in injured aging muscle and generated new muscle fiber. However, the knockdown of ITGA4 impaired their mobilization toward the site of injury, resulting in a negligent amount of new muscle growth.

### 3.4. Mechanism of ITGA4 Expression in Givi-MPCs

**Figure 7:**
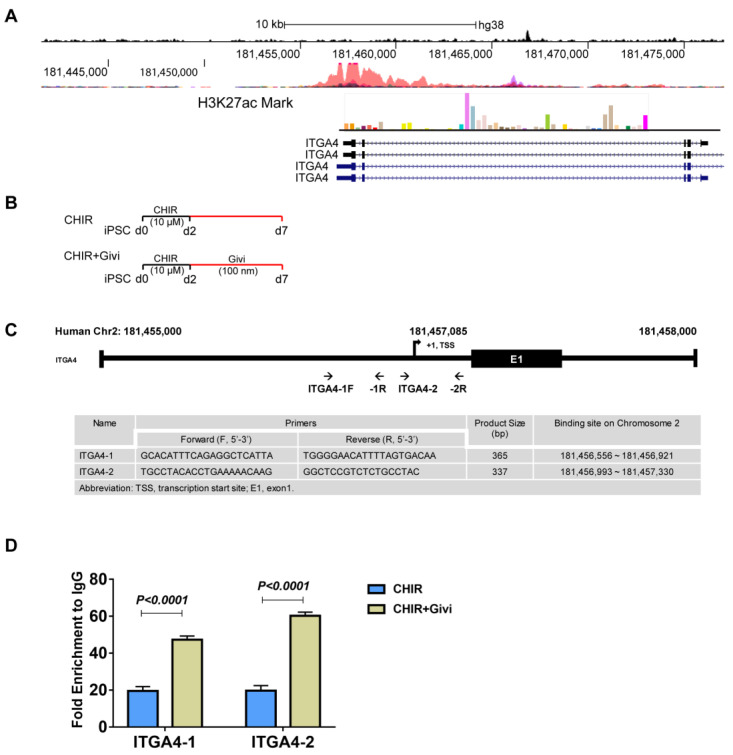
Binding activity of H3K27Ac on the ITGA4 promoter with Givinostat treatment. (**A**) H3K27ac marks at ITGA4 promoter (UCSC Genome Browser). (**B**) Schematic outline for small-molecule treatment. (**C**) Chromatin immunoprecipitation (ChIP) signals were detected by quantitative real-time PCR with IATA4 promoter-specific primers following immunoprecipitation of H3K27ac and normalized to non-immune IgG signals. (**D**) Enhanced biding activity of H3K27ac to ITGA4 promoter with Givinostat treatment compared with CHIR99021 treatment only. iPSC: induced pluripotent stem cells; CHIR: CHIR99021; Givi: Givinostat. n = 3.

Our results reveal that a high ITGA4 expression in Givi-MPCs plays an important role in homing and proliferation of MPCs in the present study. We investigated the potential mechanism of ITGA4 expression induced by Givinostat. With the UCSC Genome Browser, we found H3K27ac is a well-recognized marker at the IGTA4 promoter (Figure 7A). Next, we further performed a chromatin immunoprecipitation experiment to study whether H3K27ac augments the binding of the ITGA4 promoter to promote its transcription with Givinostat treatment. Our results showed enhanced binding activity of H3K27ac to ITGA4 promoter with Givinostat treatment compared with CHIR99021 treatment only (Figure 7B–D).

## 4. Discussion

We conclude from this study that interventions that stimulate the mobilization and engraftment of muscle progenitors show promise in the regeneration of ischemic and dystrophic muscle tissues. Regeneration is enhanced through transplanted Givi-MPCs expressing ITGA4. As a proof of concept for using MPCs in therapeutic applications, we showed that MPCs expressing specific receptors survived better in injured environments, whereas non-Givi-MPCs fared poorly [14]. Therefore, the strategy of accelerated mobilization/homing and their survival in the oxidant environment seems a promising avenue for enhancing regeneration. Cell homing constitutes an indispensable step in the repair processes of many organs [22,23]. We discovered that the CX3CL1 or VCAM-1/ITGA4 pathway mediates the inherent homing potential of MPCs. CX3CL1 or VCAM-1 are chemokines not basally expressed in ECs, but hypoxia induces their expression. Endothelial cells are thus direct targets of CX3CL1 or VCAM-1 via ITGA4, since the silencing of ITGA4 in MPCs impaired their migration or muscle regeneration. Chemotactic cytokines, or chemokines, are 8–15-kDa peptides that attract circulating ITGA4-expressing cells to peripheral tissues, where engraftment is facilitated by ITGA4 signaling. ITGA4 up-regulation on circulating MPCs favors homing to CX3CL1/VCAM-1-rich cellular sites. Indeed, recent evidence in a similar study on chemotactic legend/receptor interaction confirmed that stem-cell homing and engraftment are potently increased by DPPIV inhibition and that this improvement is negated by the small-molecule CXCR4 antagonist AMD3100 [24]. CXCR4-positive circulating cells compete for the occupancy of peripheral CXCL12-positive niches. At least one role played by CX3CL1 or VCAM-1/ITGA4 system in this study appears to direct their chemotaxis to the damaged site. Thus, it is very likely the mobilization is mediated by the ligand–receptor interaction. Collectively, our data support the hypothesis that MPCs expressing ITGA4, which sense chemokines secreted by the endothelial cells at the injury site, migrate to the damaged muscle.

Age-associated inflammation and oxidative stress in sarcopenia are major concerns for the success of muscle progenitor cell transplantation. We transplanted Givi-MPCs by intramuscular injection in the TA muscle of 18-month-old NSG mice and 16-month-old mdx/scid mice with CTX injury. Fourteen days after transplantation, improvement in muscle regeneration and reduction of fibrosis and inflammation were observed with Givi-MPC-treated mice. Even without injury, 16-month-old mdx/scid mice showed muscle necrosis, degeneration, and fibrosis. Thus, we directly injected the Givi-MPCs with H2B-EGFP reporter into the TA muscle tissue of those mice without injury to confirm whether these cells can survive and generate new muscle fibers in aged muscle. The formation of new muscle cells, detectable by EGFP signals and human dystrophin-positive cells, took place, suggesting active muscle regeneration. These data strongly support the regenerative potential of Givi-MPCs in aged muscle and sarcopenia patients with acute or chronic injury.

As discussed above, chemoattraction via CX3CL1 or VCAM-1/ITGA4 directed cellular migration to injury sites. To support this finding, we provided proof of in vivo cell-tracking through the systemic delivery of Givi-MPCs. The H2B-GFP construct provided a constitutively expressing nuclear marker for in vivo cell-tracking. With the injection of Givi-MPCs expressing H2B-EGFP reporter into the tail vein in NSG mice, a strong H2B-EGFP fluorescent nuclear signal in CTX-injured muscle tissue, not PBS-treated control muscle tissue, was detected, suggesting the mobilization of injected Givi-MPCs towards injured sites. We further investigated the potential mechanism of MPC migration for muscle growth. Givi-MPCs highly expressed ITGA4. Integrin α4 (ITGA4), a member of the integrin alpha chain family of proteins, which pairs with β1 integrin, plays a critical role during in vivo myogenesis [25,26]. ITGA4 is also involved in cell migration [25,26]. Integrin α4 subunit is expressed in the myotome and early limb muscle mass during muscle development [27,28]. Murine Lbax1+ embryonic muscle progenitors expressed ITGA4 [29]. VCAM-1 and CX3CL1 were identified as high-affinity ligands of ITGA4 [30,31,32]. The interaction between ITGA4 and VCAM-1 or CX3CL1 is involved in myogenesis, leukocyte diapedesis, and inflammation [32,33,34]. It is known that injury and inflammation up-regulate the expression of inflammatory cytokines [32,34]. In the present study, we found that TNF-α stimulation and CTX injury increased VCAM-1 and CX3CL1 expression in HAECs. These findings are consistent with previous studies that suggest the expression of VCAM-1 and CX3CL1 was induced in endothelial cells during inflammatory diseases or aging [34,35,36]. Additionally, CX3CL1 was also up-regulated in aging MuSCs [37].

To test whether the overexpression of ITGA4 in Givi-MPCs mediates the mobilization and recruitment of these cells to injury sites or inflammatory muscle tissue by interacting with VCAM-1 and CX3CL1, we silenced ITGA4 expression in Givi-MPCs. In vitro experiments showed that the silencing of ITGA4 impaired the migration of MPCs significantly toward the inflammatory endothelial cells. In parallel with in vitro data, ITGA4 silencing also blunted the homing of MPCs to injured aging muscle tissue, as demonstrated by the limited presence of HNA or EGFP-positive cells in the injured sites. Additionally, ITGA4 silencing also affected the proliferation of Givi-MPCs. In the tissue with systemically delivered ITGA4-silenced MPCs, many infiltrating mononuclear cells, and limited Myh3 or human dystrophin-positive cells, were observed in injured aging muscle. We believe that the infiltrating mononuclear cells are part of the inflammatory system, which needs to be further evaluated. However, a limited number of muscle cells with central nuclei, positive for Myh3 or human dystrophin were also observed in Givi-MPCs transfected with scramble vector. Collectively, these data support the idea that ITGA4 plays a critical role in Givi-MPCs homing to the injury site for muscle regeneration.

We further investigated the mechanism of ITGA4 expression by Givinostat. Given the fact that Givinostat is a histone deacetylase (HDAC) inhibitor, it is likely that treatment with Givinostat induced the up-regulation of ITGA4 via epigenetic modification. Interestingly, we found the acetylation of histone H3 at lysine 27 (H3K27ac) marks at the ITGA4 promoter. With the chromatin immunoprecipitation of H3K27ac analysis, we confirmed that Givinostat promoted ITGA4 transcription via H3K27ac modification.

### Limitations of the Study

Muscle loss occurs during aging and some human diseases, specifically Duchenne muscular dystrophy. Cellular therapy is considered to be a good choice to replenish muscle scar tissue. Due to the immense availability of iPSC as a cell source, cell-based therapy has become more popular in diseases associated with the loss of cells. Animal models are important tools for understanding disease mechanisms and for preclinical research of potential therapies. Immunodeficient mice are selected because of their extensive use for human cell transplantation. CTX-induced muscle injury was used in this research in the context of understanding iPSC-MPC homing, engraftment, and regeneration. These mice can be used for the preclinical testing of therapeutic interventions or to evaluate antifibrotic treatments. Instead of inducing repeated mechanical micro-injuries to mimic human muscular atrophy, the toxin was used to cause acute injury in the muscle. The resultant fibrosis could be a reliable criterion to compare muscle strength or muscle pathology with the treatment.

## 5. Conclusions

Taken together, Givi-MPCs have unique regenerative potential in aged muscles with acute injury and chronic injury. Their administration either by local or systemic delivery was equally effective, resulting in marked muscle regeneration. The CX3CL1 or VCAM-1/ITGA4 pathway mediated the inherent homing of MPCs. It is concluded that the strong migratory properties of Givi-MPCs render them an effective and desired cellular source for the treatment of sarcopenia with injury.

## Data Availability

The datasets used and/or analyzed during the current study are available from the corresponding author on request.

## Supplementary Materials

The following supporting information can be downloaded at: https://www.mdpi.com/article/10.3390/cells12141837/s1. Figure S1. Transfection efficiency was monitored by GFP signal in MPCs with shRNA or shITGA4 lentivirus vector transfection; Figure S2. (A) Representative images of H2B-EGFP iPSCs in bright field and green fluorescence protein (GFP) channel. Bar = 200 µm. (B) Representative images of muscle progenitor cells (MPCs) differentiated from H2B-EGFP iPSC cells in bright field and GFP channel. Bar = 100 µm. (C) H2B-EGFP iPSC-derived MPCs expressed transcription factor Pax7. Bar = 100 µm; Figure S3. Representative fluorescent images for transplanted cell-tracking with H2B-EGFP fluorescent signal and human nuclear antigen (HNA) from CTX-injured muscle tissue sections or PBS-treated control muscle tissue sections. Bar = 50 µm; Figure S4. H2B-EGFP-positive cells were not observed in the heart, lung, liver, kidney, and gastrocnemius (GA) muscle. Bar = 100 µm; Figure S5. Pulmonary embolism in mice with a high dosage of Givi-MPC transplantation (1 × 10^6^ cells) by tail-vein injection. EGFP-positive cells were observed in pulmonary vessels (arrows). Bar = 100 µm.
