# Supplementary material for: Chemokine/ITGA4 Interaction Directs iPSC-Derived Myogenic Progenitor Migration to Injury Sites in Aging Muscle for Regeneration
Source: Cells. 2023 Jul 12;12(14):1837. doi: 10.3390/cells12141837 (PMC10378040; doi:10.3390/cells12141837)
Supplement: Supplementary file 1 [file cells-12-01837-s001.zip › cells-2470881-supplementary.pdf]

Supplemental Figures

Supplemental Fig 1. Transfection efficiency was monitored by GFP signal in MPCs with shRNA or shITGA4 lentivirus vector transfection.

Supplemental Fig 2. (A) Representative images of H2B-EGFP iPSCs in bright field and green fluorescence protein (GFP) channel. Bar=200 µm. (B) Representative images of muscle progenitor cells (MPCs) differentiated from H2B-EGFP iPSC cells in bright field and GFP channel. Bar=100 µm. (C) H2B-EGFP iPSC derived MPCs expressed transcription factor Pax7. Bar=100 µm.

Supplemental Fig.3. Representative fluorescent images for transplanted cell tracking with H2B-EGFP fluorescent signal and human nuclear antigen (HNA) from CTX injured muscle tissue sections or PBS treated control muscle tissue sections. Bar=50 µm.

Supplemental Fig.4. H2B-EGFP positive cells were not observed in heart, lung, liver, kidney, and gastrocnemius (GA) muscle. Bar=100 µm.

Supplemental Fig.5. Pulmonary embolism in mice with high dosage of Givi-MPCs transplantation (1×10^6 cells) by tail vein injection. EGFP positive cells were observed in pulmonary vessels (arrows). Bar=100 µm.

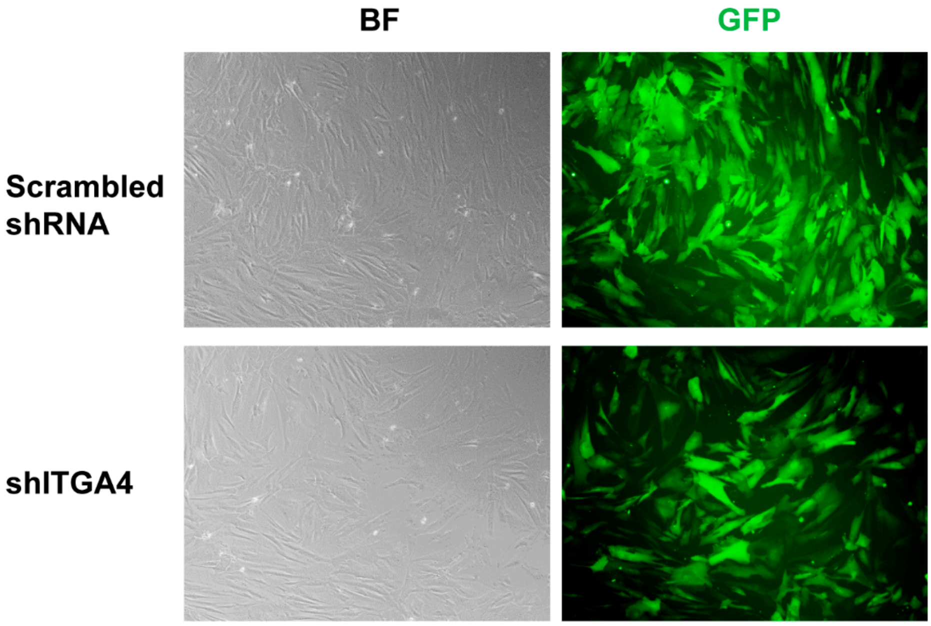

Suplemental Figure S1

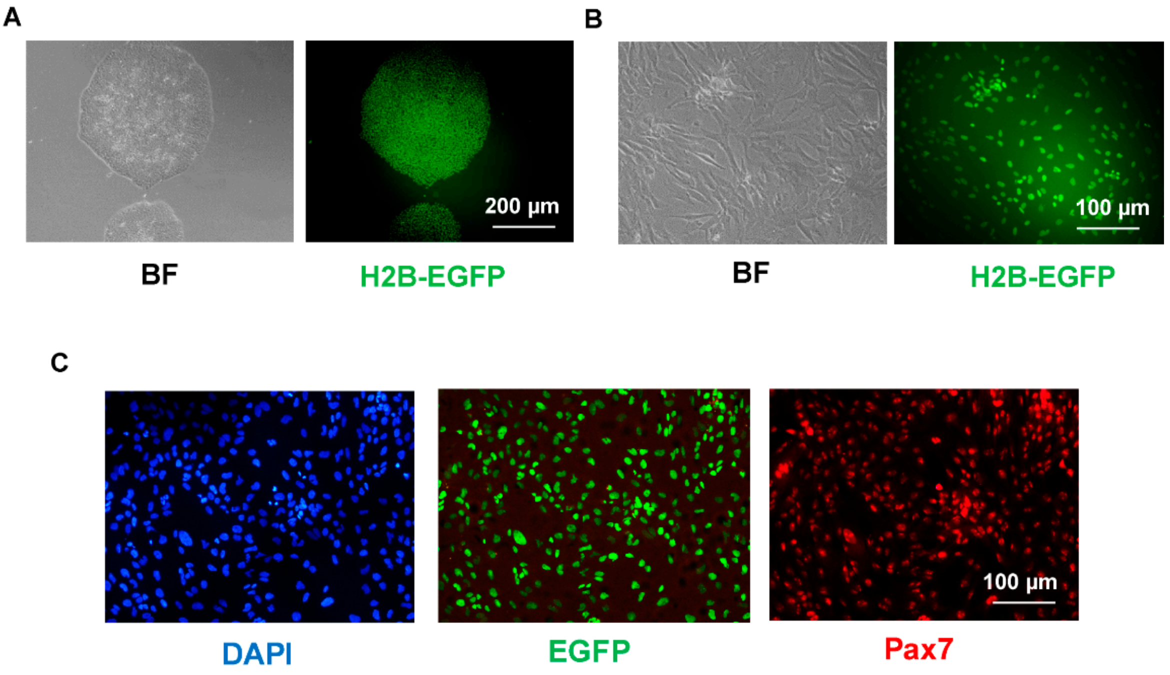

Suplemental Figure S2

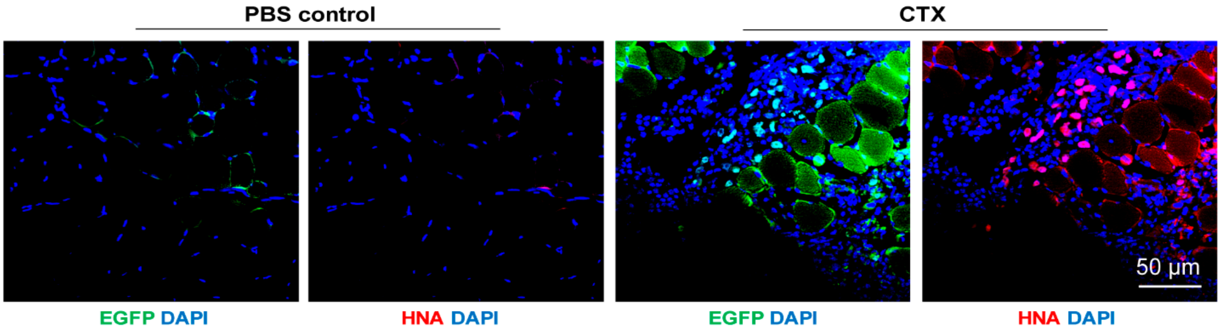

Suplemental Figure S3

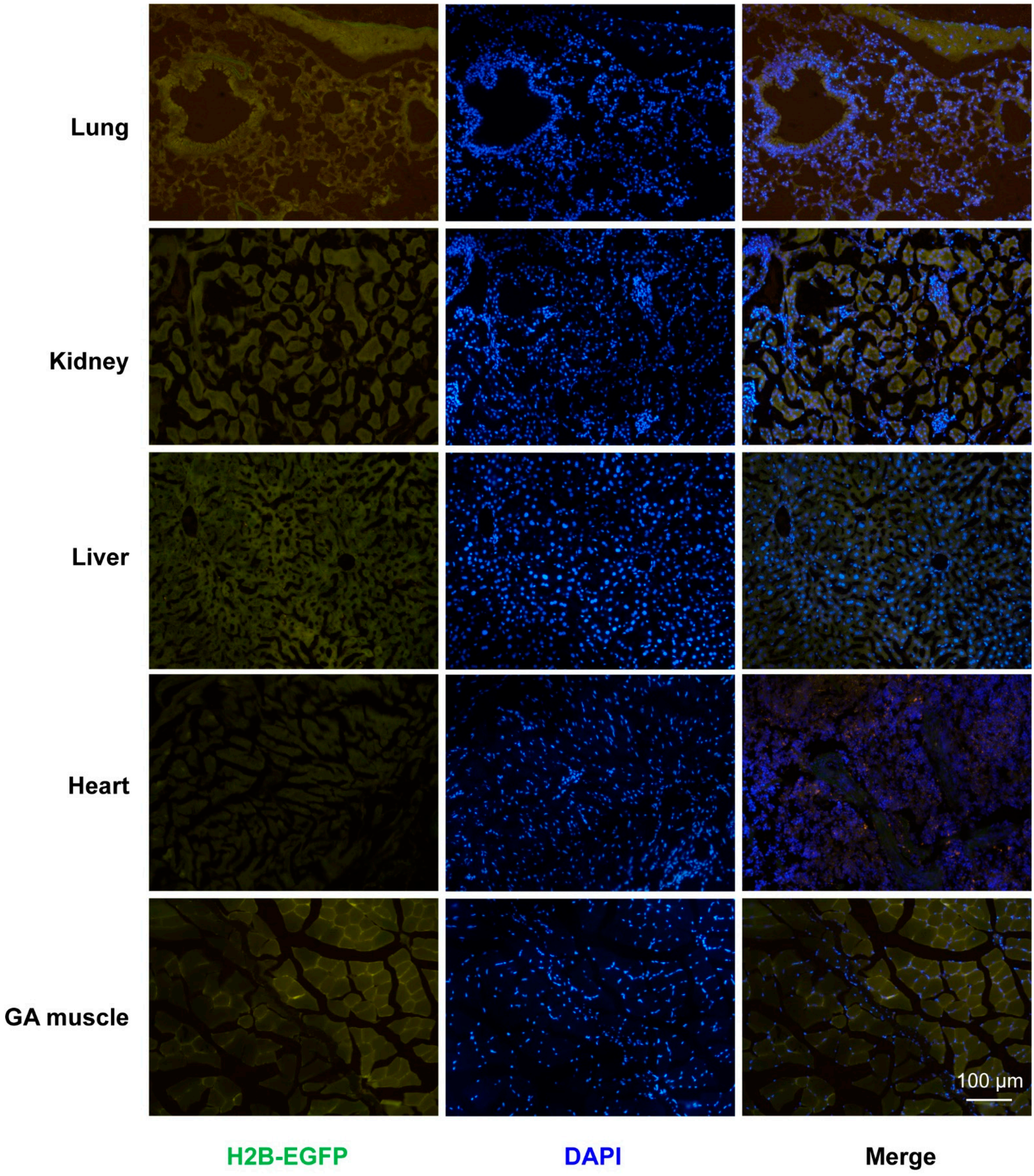

Suplemental Figure S4

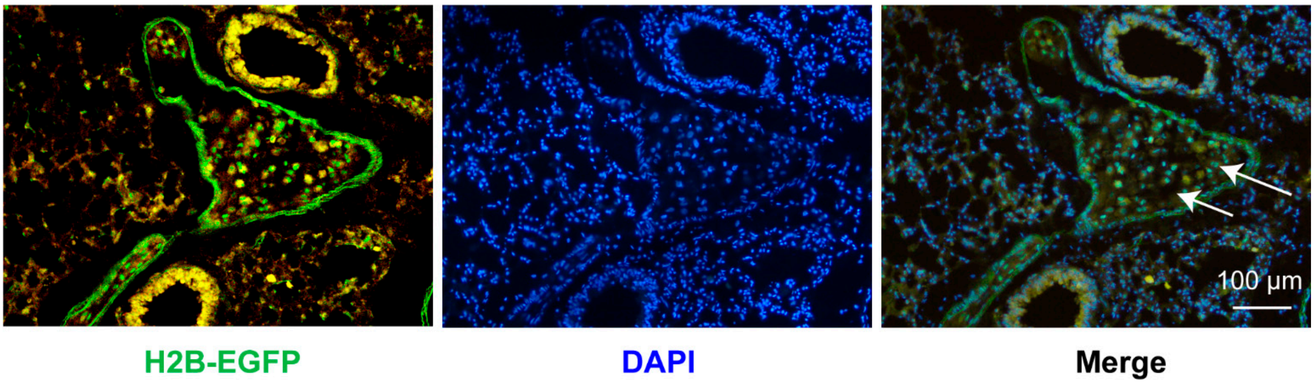

Suplemental Figure S5
